# Authors’ response: Efficacy and effectiveness of COVID-19 vaccines against SARS-CoV-2 infection: More data on asymptomatic SARS-CoV-2 infection

**DOI:** 10.2807/1560-7917.ES.2021.26.35.2100832

**Published:** 2021-09-02

**Authors:** Thomas Harder, Judith Koch, Sabine Vygen-Bonnet, Wiebe Külper-Schiek, Antonia Pilic, Sarah Reda, Stefan Scholz, Ole Wichmann

**Affiliations:** 1 Robert Koch Institute, Berlin, Germany

**To the editor**: We would like to thank the authors of the letter for their valuable comments on our article [1]. We would like to respond briefly to each point. 

In the post-hoc analysis of the Vaxzevria (ChAdOx1-S, AstraZeneca, Cambridge, United Kingdom) licensure trial by Emary et al. [2], the secondary outcome was named ‘asymptomatic infections and infection with unknown symptoms’. Since the outcome description was imprecise, we decided to exclude these data from our report. We agree with the authors of the letter that because of wide and overlapping confidence intervals, the relevance of Emary et al.’s findings is unclear. 

Dagan et al. [3] reported a supplementary analysis that included cases without documented symptoms. We decided not to take these results into account since the authors of this paper themselves recognised that their approach is ’an imperfect proxy for asymptomatic infections (since mild symptoms may not be documented)’. 

We agree with the authors of the letter that documents submitted to regulatory authorities are valuable sources of information until formal publications are available. In fact, we used the European Medicines Agency (EMA) report on the Janssen COVID-19 vaccine (Janssen-Cilag International, Beerse, Belgium) because of the lack of published evidence on this particular vaccine. As suggested by the authors, we will consider this approach in future updates of this living systematic review also for other vaccines. 

Finally, we are also in agreement that the widespread circulation of the SARS-CoV-2 Delta variant (Phylogenetic Assignment of Named Global Outbreak (Pango) lineage designation B.1.617.2) calls for an update of this living systematic review. We are currently in the process of summarising data on the vaccine effectiveness against this particular variant and hope to make them publicly available soon. 
